# Clinical Performance and Safety of the Optima Non-compliant Percutaneous Transluminal Coronary Angioplasty Balloon in Contemporary Percutaneous Coronary Intervention: A Multicenter Real-World Study

**DOI:** 10.7759/cureus.116826

**Published:** 2026-09-24

**Authors:** Sujeet Narain, Chirag Jagdish Parikh, Anup Ashok Pusate, Anuj Sarda, Hitendra Bhagwatkar

**Affiliations:** 1 Interventional Cardiology, Kailash Hospital, Greater Noida, IND; 2 Cardiology, Smt. S.R. Mehta and Sir K.P. Cardiac Institute, Mumbai, IND; 3 Interventional Cardiology, Suretech Hospital, Nagpur, IND; 4 Interventional Cardiology, Intima Heart and Critical Care, Nagpur, IND; 5 Cardiology, NKP Salve Institute of Medical Sciences and Research Centre and Lata Mangeshkar Hospital, Nagpur, IND

**Keywords:** coronary artery disease, non-compliant balloon, percutaneous coronary intervention, post-dilatation, ptca catheter

## Abstract

Background

Non-compliant (NC) balloon catheters are widely used during percutaneous coronary intervention (PCI) for lesion preparation and stent optimization because they allow controlled high-pressure dilation. However, real-world clinical data describing the procedural performance and short-term clinical outcomes of NC balloon catheters across diverse patient and lesion characteristics remain limited. This study evaluated the observed procedural performance, device performance characteristics, and short-term clinical outcomes associated with the use of the Optima NC non-compliant percutaneous transluminal coronary angioplasty (PTCA) balloon catheter (Translumina Therapeutics Private Limited, New Delhi, India) during routine PCI.

Methods

This multicenter, retrospective, single-arm observational study included patients aged ≥18 years who underwent PCI using at least one Optima NC PTCA balloon catheter at four cardiology centers in India between January and June 2024. Eligible patients had ≥80% coronary artery stenosis requiring PCI. The primary endpoint was procedural success, defined as successful balloon delivery, inflation, and retrieval without major procedural complications, together with achievement of final Thrombolysis in Myocardial Infarction (TIMI) grade III flow. Secondary endpoints included device success, operator-assessed device performance characteristics, and clinical outcomes through three months of follow-up.

Results

Of 216 patients screened, 196 (90.7%) were included in the final analysis, and 20 (9.3%) were excluded because of incomplete clinical or procedural information. The mean age was 59.20 ± 11.74 years, and 151 (77.0%) patients were male. Acute coronary syndrome was present in 191 (97.45%) patients, and multivessel disease in 111 (56.63%). The left anterior descending artery (LAD) was the most frequently treated vessel (112 (57.14%) patients). Procedural success was achieved in 193/196 (98.5%) procedures, while final TIMI grade III flow was achieved in 196/196 (100%) procedures. Device success was achieved in 193/196 (98.5%) procedures. No major adverse cardiac events (MACE) were reported during the index procedure or at discharge. During follow-up, one myocardial infarction (0.51%) was reported at one month, while one target lesion failure (TLF) (0.51%) and one cardiac death (0.51%) were reported at three months.

Conclusion

In this multicenter real-world cohort, the Optima NC PTCA balloon catheter demonstrated high observed procedural and device success, with favorable operator-assessed performance and few clinical events during short-term follow-up. Given the retrospective single-arm design and absence of a comparator, these findings are descriptive and do not establish comparative efficacy, superiority, or long-term safety. Prospective comparative studies with larger populations and longer follow-up are warranted to further characterize the clinical performance of the device.

## Introduction

Coronary artery disease (CAD) remains one of the leading causes of morbidity and mortality worldwide. Over the past few decades, percutaneous coronary intervention (PCI) has significantly transformed the management of CAD and has become one of the most commonly performed revascularization procedures, with more than two million procedures performed annually worldwide [1]. Continuous advances in PCI technology and procedural techniques have contributed to improvements in procedural success and clinical outcomes while reducing procedure-related complications [2]. Nevertheless, challenges remain during PCI, particularly with lesion preparation, stent delivery, and achievement of adequate stent expansion and apposition. Inadequate lesion preparation or balloon expansion may result in vessel wall injury, coronary dissection, or other procedural complications, which may subsequently contribute to myocardial infarction (MI) and other adverse outcomes [3]. Balloon catheters therefore remain an integral component of PCI, particularly for lesion preparation and post-dilation to facilitate adequate stent expansion and apposition [4].

Non-compliant (NC) balloons are widely used in contemporary PCI because they permit controlled dilation at relatively high inflation pressures [5,6]. Compared with semi-compliant balloons, NC balloons exhibit limited expansion beyond their nominal diameter, allowing more predictable balloon geometry and delivery of radial force during lesion preparation and stent optimization. These characteristics are particularly relevant in challenging coronary anatomies, including bifurcation lesions, calcified plaques, tortuous vessels, and other morphologically complex lesions [7]. Previous imaging-based studies, including studies using optical coherence tomography (OCT), have also evaluated the effects of NC balloon use on luminal dimensions and lesion expansion, supporting their role in contemporary lesion preparation and stent optimization strategies [8,9].

Despite the widespread use of NC balloons during PCI, real-world data describing the procedural performance and short-term clinical outcomes associated with individual NC balloon platforms across heterogeneous patient and lesion characteristics remain relatively limited. Available evidence has frequently been generated in selected lesion subsets or controlled clinical settings and may not fully represent routine clinical practice. Evaluation of device performance in real-world practice is therefore relevant, particularly in patients presenting with acute coronary syndromes and those undergoing PCI for anatomically diverse coronary lesions.

The present multicenter, retrospective, single-arm observational study aimed to describe the procedural performance, device performance characteristics, angiographic outcomes, and short-term clinical outcomes associated with the use of the Optima NC non-compliant percutaneous transluminal coronary angioplasty (PTCA) balloon catheter (Translumina Therapeutics Private Limited, New Delhi, India) during routine PCI practice in India. The study assessed procedural and device success, operator-assessed device performance characteristics, angiographic findings, procedural complications, and clinical outcomes during follow-up of up to three months after the index PCI. Given the observational, single-arm design, the study was intended to provide descriptive real-world evidence regarding the use and short-term performance of the device rather than to establish comparative effectiveness or causal device-specific clinical benefit.

## Materials and methods

Study design and setting

This retrospective, post-marketing, multicenter, single-arm observational study was conducted at four cardiology centers in India between January and June 2024. The study described the procedural performance and short-term clinical outcomes associated with the use of the Optima NC non-compliant percutaneous transluminal coronary angioplasty (PTCA) balloon catheter during PCI for coronary artery disease (CAD). The study protocol was approved by the Institutional Ethics Committees of all participating centers and was conducted in accordance with the Declaration of Helsinki, International Council for Harmonisation Good Clinical Practice (ICH-GCP) guidelines [10], and the Indian Council of Medical Research (ICMR) National Ethical Guidelines (2017) [11]. As the study involved retrospective review of anonymized clinical records, the requirement for written informed consent was waived by the respective ethics committees.

Selection Criteria

Patients aged ≥18 years who underwent PCI using at least one Optima NC PTCA balloon catheter during the index procedure were eligible. Target lesions included de novo or restenotic lesions in native coronary arteries or bypass grafts with ≥80% diameter stenosis determined by visual estimation or quantitative coronary angiography (QCA). Patients were excluded if they were pregnant or breastfeeding, had cardiogenic shock, hemodynamic instability, active infection including SARS-CoV infection, or abnormal platelet counts (<100,000 cells/mm³ or >700,000 cells/mm³) at the time of the procedure. Of 216 patients screened, 196 were included in the final analysis; 20 patients were excluded because the available records did not contain sufficient clinical information for complete study data extraction. The patient selection process is shown in Figure 1.

**Figure 1:**
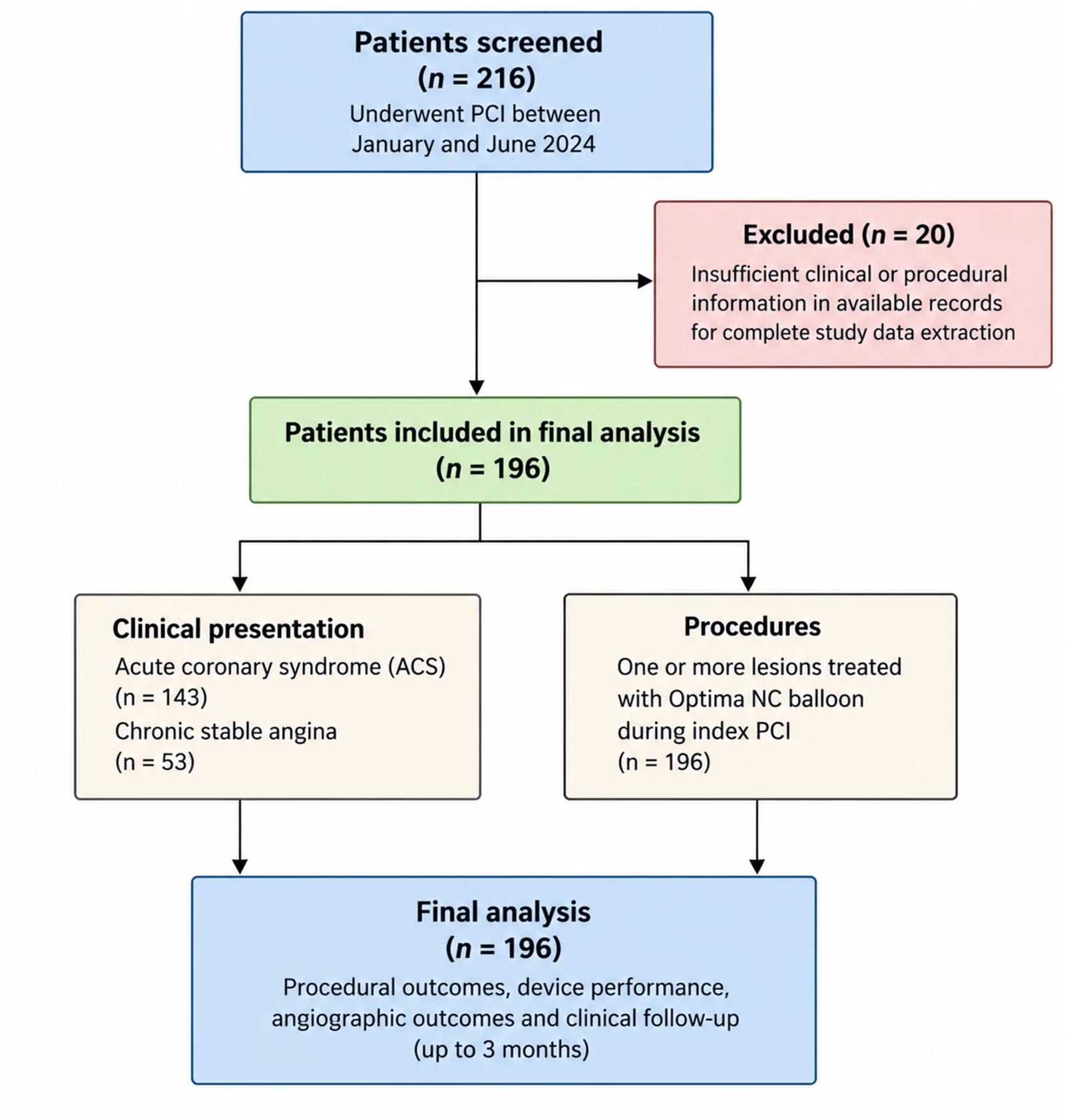
Patient selection flow diagram ACS: acute coronary syndrome, NC: non-compliant, PCI: percutaneous coronary intervention Author-generated figure prepared using Microsoft PowerPoint (Microsoft Corporation, Redmond, WA)

The patient/index PCI procedure was the primary unit of analysis. Patients with multivessel disease could have more than one treated lesion or require more than one balloon during the index procedure; therefore, lesion- and device-level observations were not considered independent patient observations.

Sample Size

As this was a retrospective post-marketing observational study, no prospective sample size calculation was performed. All eligible patients treated with the Optima NC PTCA balloon during the predefined study period with sufficient clinical and procedural information were included. The final sample comprised 196 patients. The observed procedural success rate was 98.5% (193/196; 95% confidence interval (CI): 95.6-99.5%).

Study device and outcome measures

The Optima NC PTCA balloon catheter is a CE-approved, dual-lumen rapid-exchange non-compliant balloon system designed for controlled high-pressure dilation during PCI. The device is available in diameters ranging from 1.50 to 4.50 mm and balloon lengths from 6 to 30 mm.

The primary endpoint was procedural success, defined as successful balloon delivery, inflation and deflation, and retrieval without major procedural complications, together with achievement of final Thrombolysis in Myocardial Infarction (TIMI) grade III flow. Major procedural complications included coronary perforation, flow-limiting dissection, or impaired coronary blood flow.

Secondary endpoints included device success, angiographic success, operator-assessed device performance, procedural complications, and short-term clinical outcomes. Device performance was assessed by the treating operator for pushability, trackability, crossability, marker visualization, balloon compliance, and balloon deflation. Device success required successful completion of the intended balloon procedure without a device-related complication.

Major adverse cardiac events (MACE) were defined as cardiac death, myocardial infarction (MI), or target lesion failure (TLF). TLF comprised cardiac death related to the target vessel, target vessel MI, or clinically driven target lesion revascularization. MI and cardiac death were identified according to the clinical diagnosis documented in the available medical records. Device-related complications included balloon rupture, inability to inflate, deflate, or retrieve the catheter, catheter damage or entrapment, coronary perforation, flow-limiting dissection, or other documented events considered related to the device. Clinical events were identified through retrospective review of hospital and follow-up records.

Data sources and variables

Data were retrospectively obtained from hospital medical records, catheterization laboratory records, procedural documentation, angiographic reports, and available follow-up records. Variables included demographic characteristics, cardiovascular risk factors, clinical presentation, lesion and angiographic characteristics, balloon characteristics, inflation pressure, inflation cycles, pre- or post-dilation use, procedural and device success, final TIMI flow, device performance characteristics, procedural complications, and clinical outcomes.

Follow-up was assessed for up to three months after the index procedure. Patients without available follow-up information were not considered event-free solely on the basis of missing documentation.

Statistical analysis

Statistical analyses were performed using SPSS version 26.0 (IBM Corp., Armonk, NY). Categorical variables were expressed as frequencies and percentages, while continuous variables were summarized as mean ± standard deviation (SD). Descriptive statistics were used to summarize baseline characteristics, lesion and procedural characteristics, device performance, procedural outcomes, and follow-up clinical events. Ninety-five percent confidence intervals were calculated for the primary procedural success proportion. No imputation was performed for missing data.

## Results

A total of 216 patients who underwent PCI using the Optima NC balloon catheter between January and June 2024 were screened. Of these, 20 patients were excluded because of incomplete clinical or procedural information, and 196 patients were included in the final analysis (Figure 1). Clinical follow-up was performed for the included patients, with a mean follow-up duration of 2.63 ± 0.37 months. Baseline demographic and clinical characteristics are presented in Table 1. The mean age was 59.20 ± 11.74 years, and 151 patients (77.0%) were male. Diabetes mellitus was present in 96 (48.97%) patients, hypertension in 86 (43.87%), and previous myocardial infarction in 79 (40.30%).

**Table 1:** Baseline demographic and clinical characteristics of the study population Data are presented as number (%) or mean ± SD. BP: blood pressure, CABG: coronary artery bypass grafting, CAD: coronary artery disease, MI: myocardial infarction, PCI: percutaneous coronary intervention, SD: standard deviation

Variable	Value (n = 196)
Age, years	59.20 ± 11.74
Male sex	151 (77.0%)
Heart rate, bpm	81.50 ± 17.17
Systolic BP, mmHg	138.56 ± 25.12
Diastolic BP, mmHg	83.14 ± 12.23
Diabetes mellitus	96 (48.97%)
Hypertension	86 (43.87%)
Dyslipidemia	40 (20.40%)
Smoking	36 (18.36%)
Previous MI	79 (40.30%)
Previous PCI	44 (22.44%)
Previous CABG	3 (1.53%)
Family history of CAD	13 (6.63%)

The majority of patients presented with acute coronary syndrome (191/196, 97.45%), including unstable angina in 91 (46.43%), ST-segment elevation myocardial infarction (STEMI) in 83 (42.35%), and non-ST-segment elevation myocardial infarction (NSTEMI) in 17 (8.67%). Chronic stable angina was present in 5 (2.55%) patients. Multivessel coronary artery disease was present in 111 (56.63%) patients, while 85 (43.37%) had single-vessel disease. The left anterior descending artery (LAD) was the most frequently treated vessel (112, 57.14%), followed by the right coronary artery (RCA) (62, 31.63%) and left circumflex artery (LCX) (21, 10.71%). Detailed lesion and angiographic characteristics are presented in Table 2.

**Table 2:** Lesion and angiographic characteristics Data are presented as number (%) or mean ± SD. ACC/AHA: American College of Cardiology/American Heart Association, IVUS: intravascular ultrasound, LAD: left anterior descending artery, LCX: left circumflex artery, LMCA: left main coronary artery, OCT: optical coherence tomography, RCA: right coronary artery, STEMI: ST-segment elevation myocardial infarction, NSTEMI: non-ST-segment elevation myocardial infarction, SD: standard deviation, TIMI: Thrombolysis in Myocardial Infarction

Parameter	n (%) / Mean ± SD
Acute coronary syndrome	191 (97.45%)
Unstable angina	91 (46.43%)
STEMI	83 (42.35%)
NSTEMI	17 (8.67%)
Chronic stable angina	5 (2.55%)
Single-vessel disease	85 (43.37%)
Multivessel disease	111 (56.63%)
LAD	112 (57.14%)
RCA	62 (31.63%)
LCX	21 (10.71%)
LMCA	1 (0.51%)
Diameter stenosis, %	90.74 ± 6.76
Lesion length, mm	23.68 ± 7.89
Minimum lumen diameter, mm	1.89 ± 0.83
Reference vessel diameter, mm	2.69 ± 0.40
Bifurcation lesion	26 (13.20%)
TIMI 0	2 (1.02%)
TIMI I	47 (23.98%)
TIMI II	35 (17.86%)
TIMI III	112 (57.14%)
De novo lesion	183 (93.40%)
Total occlusion	2 (1.02%)
Thrombus present	29 (14.79%)
ACC/AHA Type A	36 (18.36%)
ACC/AHA Type B	93 (47.44%)
ACC/AHA Type C	67 (34.20%)
Eccentric plaque	115 (58.67%)
Concentric plaque	81 (41.33%)
Calcified plaque	38 (19.38%)
Non-calcified plaque	158 (80.62%)
IVUS/OCT use	12 (6.12%)

Baseline diameter stenosis was 90.74 ± 6.76%, with a mean lesion length of 23.68 ± 7.89 mm. Bifurcation lesions were present in 26 (13.20%) cases. Most lesions were de novo (183, 93.40%), while ACC/AHA type B and type C lesions accounted for 93 (47.44%) and 67 (34.20%), respectively. Calcification was present in 38 (19.38%) lesions, and IVUS/OCT was used in 12 (6.12%) cases.

The Optima NC balloon was used predominantly for post-dilation (151, 77.0%), while pre-dilation was performed in 45 (23.0%) cases. The mean balloon diameter was 3.00 ± 1.02 mm, the mean balloon length was 12.01 ± 3.11 mm, and the mean inflation pressure was 18.63 ± 3.67 atm. Procedural success and device success were achieved in 193 of 196 (98.5%) cases. Final TIMI III flow was achieved in all 196 (100%) cases. Thus, three cases did not meet the predefined composite criteria for procedural/device success despite achieving final TIMI III flow. No major adverse cardiac events were reported during the index procedure or before discharge.

During clinical follow-up, one myocardial infarction (0.51%) was reported at one month, while one case each of target lesion failure (0.51%) and cardiac death (0.51%) was reported at three months. Clinical follow-up was performed during the scheduled follow-up period, with the reported clinical events observed during follow-up. The balloon, procedural, and clinical outcome characteristics are presented in Table 3.

**Table 3:** Balloon, procedural and clinical outcome characteristics Data are presented as number (%) or mean ± SD. MI: myocardial infarction, SD: standard deviation, TIMI: Thrombolysis in Myocardial Infarction, TLF: target lesion failure

Parameter	Value
Balloon characteristics	
Balloon diameter, mm	3.00 ± 1.02
Balloon length, mm	12.01 ± 3.11
Inflation pressure, atm	18.63 ± 3.67
Inflation cycles	2.56 ± 1.27
Procedural characteristics	
Device used for pre-dilation	45 (23.0%)
Device used for post-dilation	151 (77.0%)
Procedural success	193 (98.5%)
Device success	193 (98.5%)
Final TIMI III flow	196 (100%)
Clinical outcomes during follow-up	
Myocardial infarction	1 (0.51%)
Cardiac death	1 (0.51%)
Target lesion failure	1 (0.51%)

Operator-assessed device performance was favorable across the evaluated parameters. Pushability, trackability, and crossability were rated satisfactory in more than 99% of procedures, while balloon deflation and compliance were rated satisfactory in more than 98%. Marker visualization was rated satisfactory in 95% of procedures (Figure 2).

**Figure 2:**
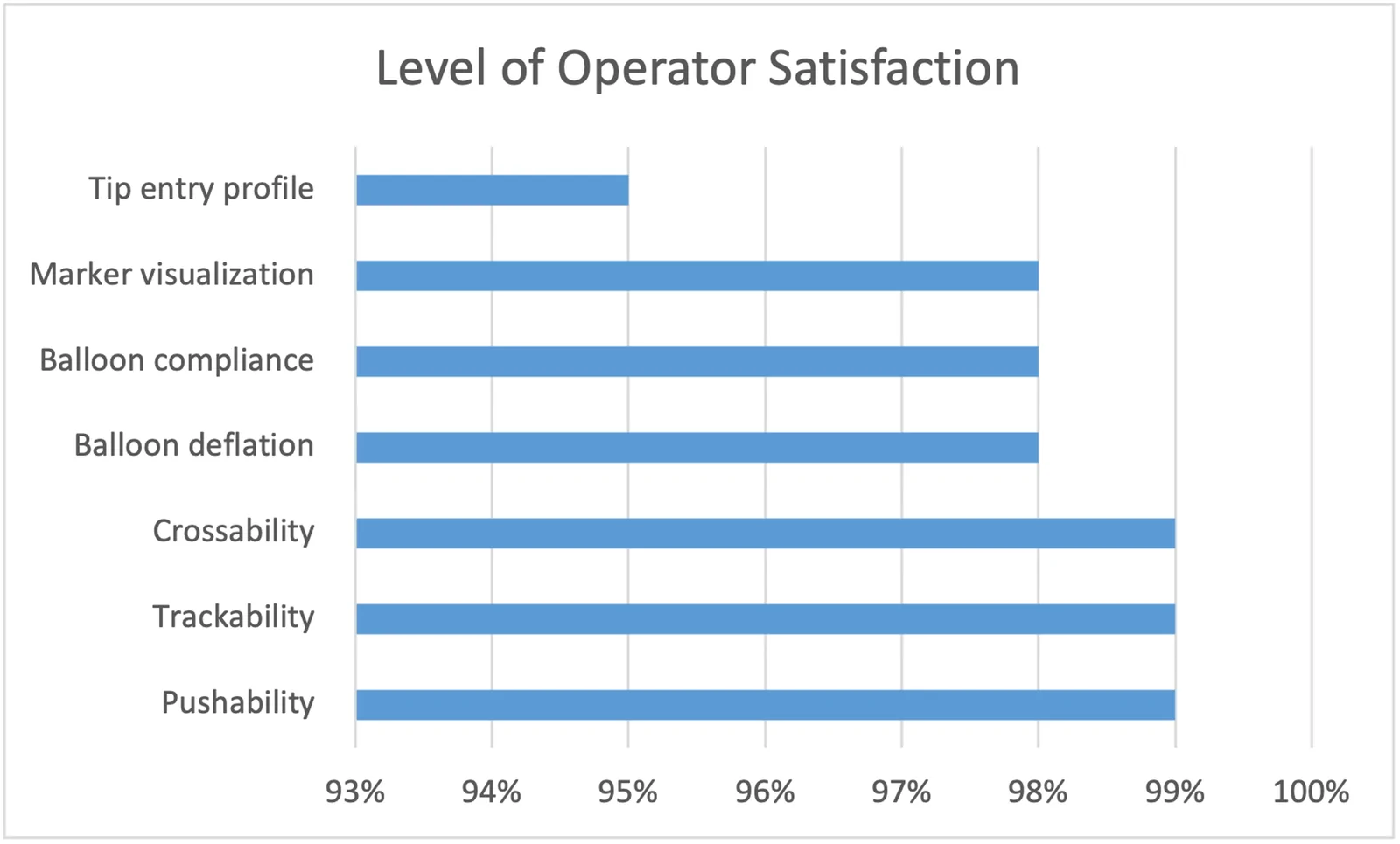
Operator-assessed Optima NC PTCA catheter parameters Operator-assessed performance of the Optima NC PTCA catheter across predefined parameters, including pushability, trackability, crossability, balloon deflation, compliance, and marker visualization. NC: non-compliant, PTCA: percutaneous transluminal coronary angioplasty Author-generated figure prepared using Microsoft PowerPoint (Microsoft Corporation, Redmond, WA)

## Discussion

The present multicenter retrospective study evaluated the observed procedural performance and short-term clinical outcomes associated with the use of the Optima NC non-compliant PTCA balloon catheter during PCI in a real-world population. The study included patients with a broad range of clinical and angiographic characteristics, with a high prevalence of acute coronary syndrome, multivessel coronary artery disease, and lesions of varying complexity. Procedural and device success were achieved in 98.5% of cases, while final TIMI III flow was achieved in all cases. These findings indicate favorable observed procedural performance of the device in routine clinical practice. However, given the retrospective single-arm design, the findings should be interpreted as descriptive and do not establish comparative efficacy or superiority over other balloon catheter platforms.

The population included several features that may increase procedural complexity, including bifurcation lesions, thrombus-containing lesions, calcified plaques, and ACC/AHA type B and C lesions. The high overall procedural success observed across this heterogeneous population provides real-world information regarding the use of the device in different clinical and anatomical settings. Nevertheless, the study was not designed to compare outcomes according to lesion complexity, and therefore, the findings should not be interpreted as demonstrating superior performance specifically in complex lesions.

The findings are broadly consistent with previous clinical evaluations of non-compliant balloon catheters. Evidence from the COBIS II registry has highlighted the role of non-compliant balloon use in PCI, particularly in the setting of coronary bifurcation interventions [12]. A clinical evaluation of the River NC® coronary balloon catheter reported high device success in a population that included patients with multivessel disease and complex coronary lesions [13]. Similarly, Pradhan et al. reported favorable procedural and device outcomes with the Mozec™ NC PTCA balloon catheter [14]. The present study adds real-world multicenter experience with the Optima NC catheter and demonstrates similarly high observed procedural and device success. However, differences in study design, patient characteristics, endpoint definitions, and procedural practices limit direct comparison of success rates across these studies.

Calcified coronary lesions remain an important challenge during PCI because they may impair lesion preparation, balloon expansion, stent delivery, and optimal stent expansion. Previous clinical data have demonstrated that moderate-to-severe coronary calcification is associated with greater procedural complexity and increased risks of adverse outcomes, including target vessel myocardial infarction and stent thrombosis in certain populations [15,16]. In the present study, calcified lesions were present in 38 (19.38%) cases, while overall procedural and device success remained high. These findings indicate that the device was used in patients with calcified lesions but do not establish superior performance in calcified anatomy. Dedicated comparative studies would be required to determine its effectiveness in this lesion subset.

The Optima NC catheter was used during both pre-dilation and post-dilation, with post-dilation accounting for the majority of procedures. Operator-assessed pushability, trackability, crossability, balloon deflation, compliance, and marker visualization were also rated favorably. These observations provide useful real-world information regarding device handling and procedural use. However, these assessments were operator-based and were not independently validated by a core laboratory or standardized objective testing; consequently, they should be regarded as descriptive measures of user experience rather than evidence of comparative technical superiority.

The predominance of post-dilation use is consistent with the established role of non-compliant balloons in stent optimization. The ability to use the same balloon platform during different stages of PCI also demonstrates its versatility within the observed cohort. However, whether this versatility translates into shorter procedure times, reduced resource utilization, improved stent expansion, or better clinical outcomes was not evaluated in the present study and should be investigated in future comparative studies.

Short-term clinical outcomes were favorable, with one myocardial infarction reported at one month and one case each of target lesion failure and cardiac death reported at three months. The low number of observed events is reassuring in the context of the present cohort, but these findings should be interpreted cautiously because of the modest sample size and short follow-up duration. The study was not designed or powered to assess uncommon adverse events or longer-term outcomes such as late restenosis, target lesion revascularization, or stent-related complications.

Overall, this multicenter real-world analysis demonstrated a high observed rate of procedural and device success and favorable operator-assessed performance of the Optima NC non-compliant PTCA balloon catheter. The findings support its use as a balloon platform in contemporary PCI practice but do not establish comparative efficacy, superiority, or long-term safety. Larger prospective comparative studies with standardized endpoint definitions, independent event adjudication, objective assessment of lesion preparation and stent expansion, and longer follow-up are warranted to further characterize the clinical performance of the device.

Strengths and limitations

This study has several strengths, including its multicenter real-world setting, inclusion of patients with diverse clinical presentations and lesion characteristics, and assessment of procedural performance together with short-term clinical outcomes. However, the retrospective single-arm design and absence of a comparator group limit causal interpretation and preclude direct comparison with other balloon catheter platforms. The sample size was modest, and the relatively short follow-up limits assessment of longer-term outcomes, including late target lesion failure and repeat revascularization. Operator-assessed device performance was subjective and may be influenced by individual experience or reporting bias. Intravascular imaging was used in only a small proportion of cases, limiting detailed assessment of lesion preparation and stent expansion. Clinical events were derived from available clinical documentation rather than independent external event adjudication. Finally, manufacturer involvement should be considered when interpreting the findings.

## Conclusions

The Optima NC PTCA balloon catheter demonstrated high observed procedural and device success and favorable operator-assessed performance in this multicenter real-world cohort undergoing PCI. The catheter was used for both pre-dilatation and post-dilatation across a broad range of coronary lesion characteristics, with few clinical events observed during short-term follow-up. However, given the retrospective single-arm design, these findings should be interpreted as descriptive and do not establish comparative efficacy, superiority, or long-term safety. Prospective comparative studies involving larger populations, standardized endpoint assessment, and longer follow-up are warranted to further evaluate the clinical performance and durability of outcomes associated with the Optima NC catheter.
